# AnnoSys—implementation of a generic annotation system for schema-based data using the example of biodiversity collection data

**DOI:** 10.1093/database/bax018

**Published:** 2017-03-18

**Authors:** L. Suhrbier, W.-H. Kusber, O. Tschöpe, A. Güntsch, W. G. Berendsohn

**Affiliations:** a Botanic Garden and Botanical Museum, Freie Universität Berlin, Königin-Luise-Str. 6-8, 14195 Berlin, Germany

## Abstract

Biological research collections holding billions of specimens world-wide provide the most important baseline information for systematic biodiversity research. Increasingly, specimen data records become available in virtual herbaria and data portals. The traditional (physical) annotation procedure fails here, so that an important pathway of research documentation and data quality control is broken. In order to create an online annotation system, we analysed, modeled and adapted traditional specimen annotation workflows. The AnnoSys system accesses collection data from either conventional web resources or the Biological Collection Access Service (BioCASe) and accepts XML-based data standards like ABCD or DarwinCore. It comprises a searchable annotation data repository, a user interface, and a subscription based message system. We describe the main components of AnnoSys and its current and planned interoperability with biodiversity data portals and networks. Details are given on the underlying architectural model, which implements the W3C OpenAnnotation model and allows the adaptation of AnnoSys to different problem domains. Advantages and disadvantages of different digital annotation and feedback approaches are discussed. For the biodiversity domain, AnnoSys proposes best practice procedures for digital annotations of complex records.

**Database URL:** https://annosys.bgbm.fu-berlin.de/AnnoSys/AnnoSys

## Introduction

### The domain context: annotations in biological collections

Biological collections serve as an archive for biological material used in scientific research. Natural history collections consist of preserved specimens of plants, algae, fungi and animals, which are used to study the variability of species and their evolutionary context, define their name (‘type specimens’), and which serve as vouchers for a wide range of research in biology and beyond. Globally, these collections contain 2–3 billions of specimens (1) going back for hundreds of years, collected all over the world, thus preserving a falsifiable record of the occurrence of a species in space and time. Examples of research applications of specimens include studies of the effect of climate change on species distribution, predictive modeling of the spread of disease vectors or pathogens, or threats posed by potentially invasive species (2).

**Figure 1.**
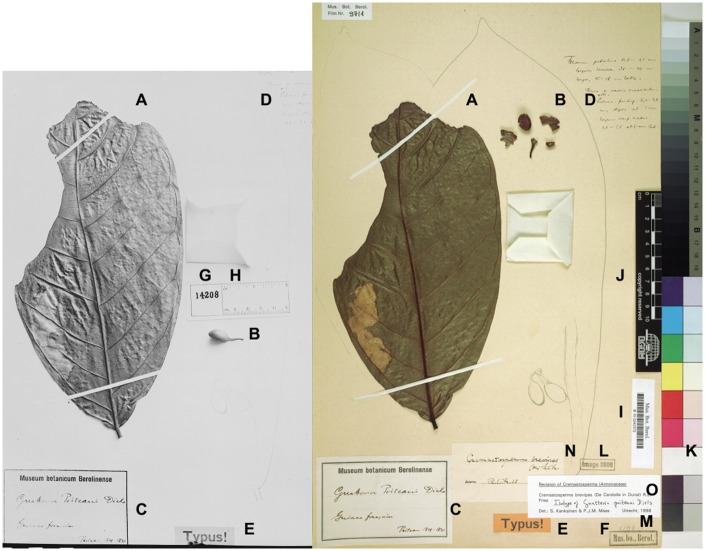
Example of traditional annotations on a herbarium specimen collected in the early 19th century: images of the same herbarium specimen (B 10 0242372) taken in the 1930s (left, identified as *Guatteria poiteaui*) and in 2006 (right, identified as *Cremastosperma brevipes*). This demonstrates the potential disconnect between the virtual specimen image (or record) and the actual object; in the future we expect that the virtual specimen will increasingly become the prime object of annotations, which can be accessed and managed with AnnoSys. (**A**) Leaf mounted on the herbarium sheet; (**B**) fruit (dissected on the right); (**C**) original herbarium label; (**D**) handwritten early annotation including additional morphological details (from duplicates?); (**E**) label indicating the type (name-giving) status of the specimen; (**F**) property-indicating stamp of the Berlin herbarium (cut off on the left); (**G**, **H**) ephemeral photographic negative number and scale bar; (**I**) permanent barcode label (UUID); (**J**) permanent scale bar; (**K**) ephemeral colour chart; (**L**) stamp indicating digitisation; (**M**) (handwriting): internal documentation of a loan; (**N**) annotation label as of 1938; (**O**) annotation as of 1998. Source (left image): The Field Museum of Natural History (2014). J. F. Macbride's Historical Photographs (1929–39) of Type Specimens from Berlin (B) (CC BY-NC 4.0); (right image): Röpert D. (ed.) 2000 + (continuously updated): Digital specimen images at the Herbarium Berolinense.—Stable identifier: http://herbarium.bgbm.org/object/B100242372 (CC BY-SA 3.0) (accessed 9 June 2016).

When studying these objects, researchers traditionally add annotations to the physical specimens, thus improving the quality of the associated data (3) (e.g. the taxonomic identification—the species name assigned to the specimen and thus to the sampled organism—see example in Figure 1). Increasingly, traditional research workflows involving loans or visits to the host institution by the researcher are complemented by studies in virtual collections with specimen data and images made available through the Internet. A user-friendly, general online mechanism enabling researchers, particularly taxonomists, to annotate virtual specimens beyond simple text email comments is thus needed.

With this challenge in mind, we first analysed classical annotation workflows (e.g. in herbaria) as well as available digital annotation systems in the biodiversity domain. The features implemented by the SYNTHESYS annotation system (4) turned out to be closest to our aims. The SYNTHESYS workflow was based on the infrastructure of the Global Biodiversity Information Facility (GBIF) (http://www.gbif.org/) (5) and used community data standard XML (http://www.w3.org/TR/xml11) schemas like Access to Biological Collection Data [ABCD] (6) or DarwinCore (http://rs.tdwg.org/dwc/index.htm) as well as agreed data access protocols such as BioCASe (http://www.biocase.org), DiGIR (http://digir.sourceforge.net/), and TAPIR (http://www.tdwg.org/standards/449). For the AnnoSys prototype and initial release, we chose ABCD and the BioCASe protocol.

Biological Collection Access Services (BioCASe) (http://www.biocase.org) is a transnational network of diverse biological collections that provides unified access to distributed and heterogeneous collection and observational databases using open data standards and protocols. The BioCASe Provider Software (http://www.biocase.org/products/provider_software/) is an xml data binding middleware representing an abstraction layer in front of a database. The software translates BioCASe requests into database queries and wraps the result into standard XML documents (e.g. ABCD) according to a mapping to the local database schema. BioCASe thus allows standard access to a variety of database management systems and arbitrarily structured databases.

Experience from earlier systems (7) showed that data records obtained from providers change over time. Thus, an annotation system cannot rely on the availability of the exact record which had been provided when the annotation has been created (the original record) from data providers. The main reason is that generally provider databases lack a revision management or archiving system making historic data records persistently accessible. As a consequence, an annotation directly referring to the provider’s data record may be invalid, because that data record has changed and does not contain the originally annotated information. A second important fact drawn from experience was that data providers are often not able to instantly update their records when corrections were communicated to them (e.g. by the private e-mail feedback mechanism mediated by the GBIF secretariat). In some cases, such as data taken from print publications, direct correction of the source data is even undesirable.

For these reasons, original data records have to be stored together with the annotations and both have to be made publicly accessible. This formed the baseline for the AnnoSys project (https://annosys.bgbm.fu-berlin.de/).

### Annotation tools and annotation servers in other domains

Annotation tools and servers are starting to become increasingly common. Examples for web annotation tools are Hypothes.is (https://hypothes.is/), Domeo (http://www.hcklab.org/annotation-domeo.html) (8), Pundit (http://thepund.it), the Homer Multitext Project (http://www.homermultitext.org/hmt-doc/cite/index.html) or the CATCH project (https://github.com/annotationsatharvard/catcha), some of them being developed in parallel to or after the development of AnnoSys. However, these annotation tools differ from the AnnoSys approach in that they mainly aim at annotating web-resources like web pages, documents, images or other multimedia objects, using free text comments, (semantic) tags or references to other resources. AnnoSys focuses on annotating specific elements of single or batches of structured data records. Although the Morphbank annotation tool (9) also allows users the creation of structured annotations to biological specimens, it is restricted to the Morphbank system and does not provide annotations of record data from external resources or data providers. This topic is addressed by the annotation tool SharedCanvas (10), which facilitates the interoperability of repositories of culturally important handwritten documents (11) by allowing to describe the interrelationships of resources like texts or images, but SharedConvas does not allow for simultaneous structured annotations. To enable this functionality for our current use case ‘specimen data’, data portals would be required to add e.g. RDFa microtags (http://www.w3.org/TR/rdfa-primer/) to their HTML-output in order to enable correct mapping of displayed information to specific elements of structured data records. Currently, this is beyond the resources of most data portals in our domain. Consequently, in AnnoSys the relationship between displayed information and structured data is realised through XML documents shared by the use of data from the original providers.

Examples for annotation servers are Annotopia (https://github.com/Annotopia) or the Lorestore (http://openannotation.metadata.net/lorestore) annotation server. Both can be seen as early models for the current release candidate of the W3C Web Annotation Protocol (https://www.w3.org/TR/annotation-protocol/), as it already provides storage and search capabilities for Open Annotation (OA) compatible annotations via a REST API interface. Most recently, Anno4j (12) provides a Java API which is also compliant with the release candidate W3C Web Annotation Model.

In this paper, the implemented technical solution that form the outcome of the past 4 years of work is described and the underlying theoretical base is presented, with special attention given to the integration of community data standards with the forthcoming W3C OA standard.

## Workflows and implemented system architecture

### User workflow

**Figure 2.**
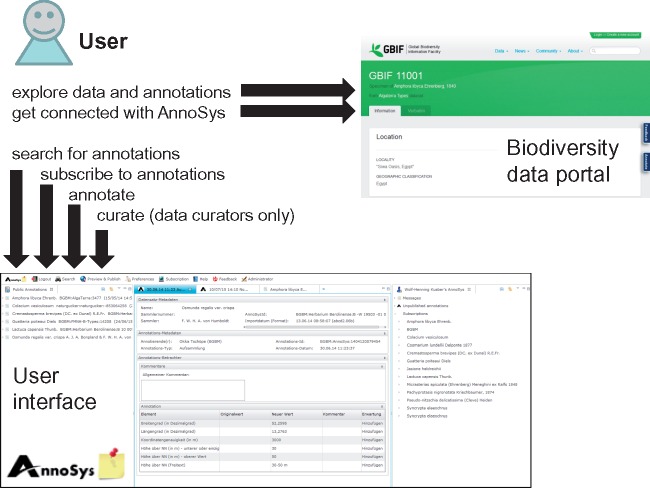
Simplified AnnoSys system workflow. Users access annotations via biodiversity data portals or directly via the AnnoSys user interface. Annotations are publicly visible in the data portals directly after publication.

The currently implemented workflow is shown in Figure 2. A list of data portals already integrating AnnoSys is given in Table 1. Clicking the ‘annotate’ link in the record view of one of these portals refers users to the AnnoSys server, where they can add an annotation. In contrast to earlier models of the workflow (3), login/registration is now only required when actually creating annotations, so that there is no barrier for users accessing and querying published annotation records. For login, a one-time registration with full name, institution, user name (login id), password and email address is necessary, as well as the acceptance of AnnoSys’ terms of use (https://annosys.bgbm.org/terms-of-use).

**Figure 3.**
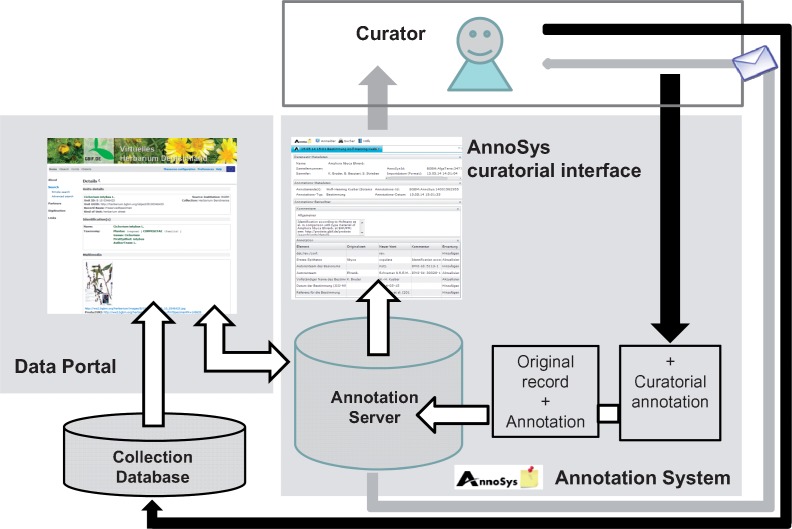
AnnoSys workflow. White arrows: user actions; dark gray arrows: data flow; light gray arrows: mailing system.

**Table 1.** Biodiversity portals integrating AnnoSys.

Name	URL	Scope
GBIF	http://www.gbif.org/	Worldwide biodiversity data
GGBN	http://www.ggbn.org	Worldwide DNA and tissue samples and related biodiversity data
EDIT Specimen and Observation Explorer for Taxonomists^a^	http://search.biocase.org/edit	Worldwide biodiversity data
World Flora Online Specimen Explorer for Phytotaxonomists	http://wfospecimens.cybertaxonomy.org/	Worldwide botanical specimen data
BioCASE, Biological Collection Service for Europe^a^	http://search.biocase.org/europe	Biodiversity data of Europe
BiNHum Sammlungsportal des Humboldt-Rings	http://binhum.net/	Portal of Collections of institutions of the Humboldt Ring, Germany
VH/de German Virtual Herbarium	http://vh.gbif.de	Portal of German Herbaria in Germany
GBIF Deutschland Botanik	http://search.biocase.de/botany	Biodiversity data of Germany, AnnoSys implemented
GBIF.DE Algae and Protists^a^	http://protists.gbif.de	Worldwide biodiversity data of algae and protists
Herbarium Berolinense—Virtual Herbarium	http://ww2.bgbm.org/herbarium/default.cfm	Digitised herbarium data at B
BIOCASE portal for BGBM collection	http://search.biocase.org/bgbm	Specimen data of all collections of the BGBM
JACQ	http://herbarium.univie.ac.at/database/search.php	Herbarium specimen management system, used by 30 institutions

a Currently only for ABCD records.

Adding an annotation starts by selecting one of the predefined annotation types (see Table 2). After data entry, the annotation data are stored together with the related version of the original record. Users can publish the annotation, so that it becomes available to the public and the AnnoSys message system informs subscribers about the annotation event. As part of ‘good scientific practice’, name and institutional affiliation of the annotator become visible with the published annotation record, mimicking the practice in annotation labels for physical specimen objects.

**Table 2.** Implemented filter types for specimen records in queries and for notifications.

Topic	Content
Species	Identification: scientific name of the species, comprising genus name and species epithet
Genus	The genus part of the scientific name
Family	Name of the family the scientific name is assigned to
Collector name	Name of a person or a team who collected the specimen
Collector’s number	(Field) number given by collector to the specimen
Country	Name of the country where the specimen was collected
Institution code	Publishing institution
Collection code	(Sub-) collection within the institution
Catalogue number	Specimen’s identifier
Identified by	Name of person who identified the specimen
Annotator	Annotator’s name

Another means to access the AnnoSys system is by way of the ‘show annotations’ functionality in the referring data portal. Following that AnnoSys link refers users to AnnoSys’ Annotation View, where the selected annotation is displayed in detail. This view is also directly accessible via the AnnoSys query interface, which helps users to find annotations matching a given set of filter criteria referring to the specimen data (see Table 3) and/or to the annotating person. Another feature added to the workflow in the current release (https://annosys.bgbm.fu-berlin.de/AnnoSys/AnnoSys) is the curatorial functionality. It is tailored for AnnoSys workflows, but generalizable for structured data with an underlying defined schema. Curators of a collection can request and be assigned curatorial rights, i.e. they can annotate annotations referring to their own collection objects. In that case, AnnoSys informs annotators and subscribers (via the message system) that a specific annotation has been accepted or rejected by the collection curator and, where applicable, that the annotation is incorporated in the underlying data base

**Table 3.** Basic Annotation Types.

Annotation type	Content	Elements involved
Determination	Elements used when an organism is identified or its identification is revised	Full scientific name, identification made by, identification date, reference URI etc.
Gathering	Elements used to describe the collection event and the locality where a specimen has been collected	Collector, collector’s field number, locality, country, latitude, longitude, altitude, date etc.
Nomenclatural type	Elements referring to the name bearing specimen of an organism	Type status, full scientific name, person assigning the type status, reference, URI etc.
Scientific name	Elements to orthographically correct a given scientific name	Full scientific name, genus, first epithet, infraspecific epithet, author, higher taxon name etc.

(Figure 3).

Ideally, a full chain of process steps in the AnnoSys workflow would be: (i) a record is annotated by a user, (ii) the annotation becomes visible in all portals connected with AnnoSys that display the same record, (iii) subscribed users and the curator responsible for the record are informed about the annotation, (iv) the curator annotates the annotation by publishing a curatorial annotation which states whether the annotation is rejected—or accepted and will therefore be incorporated into the collection database, (v) again, this curatorial annotation is visible in all portals that integrate with AnnoSys and display the same record, (vi) subscribed users are informed via the message system about the curatorial annotation and (vii) eventually the annotation is deprecated if it has been incorporated at the source. As mentioned before, this idealised process chain does not apply to all kinds of data, and limited institutional resources may delay the process considerably—in that case the AnnoSys repository acts as the knowledge store that allows for asynchronous processing of the information.

AnnoSys handles independent changes in data content by checking whether the retrieved XML-document that is to be annotated has different (older) versions in the repository. If this is the case, then the older version is flagged as a ‘historic version’ and cannot be annotated any more (see section Persistent AnnoSys Identifiers below).

A future extended curatorial workflow may include automated update mechanisms for specific collection management systems. More detailed information on the AnnoSys interface and what users can do in it can be gained from the AnnoSys Portal, which is openly accessible at https://annosys.bgbm.fu-berlin.de/AnnoSys/AnnoSys.

### General overview of the system architecture

**Figure 4.**
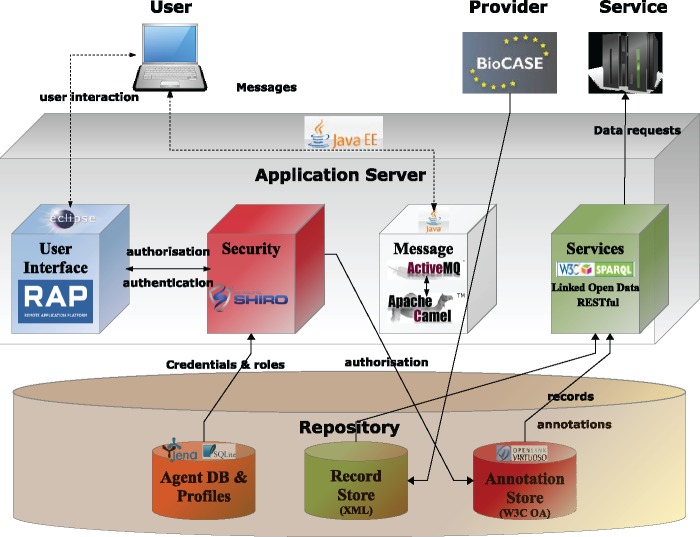
Overview of the AnnoSys system architecture.

Figure 4 provides an overview of AnnoSys’ system architecture including all components and their main information flows. The main components are briefly described below. For more detailed information, please refer to the project’s technical documentation (http://wiki.bgbm.org/annosys/index.php/TechnicalDocumentation).

### User interface

As a side line to their on-going specimen-based research, scientists have to accurately annotate or comment on specific data elements, and data curators may change their data bases according to the annotation or reject it. All these activities need to be documented and communicated back to annotators and other subscribing members of the community.

The user interface component should guide users through all these workflows. It thus must present the information clearly, be adaptable to individual user preferences, and adopt the look and feel of desktop applications users are acquainted with. The current user interface is based on usability testing and user feedback, but we expect further adaptations with wider use in online information systems (Table 1).

The implementation of the AnnoSys user interface is based on Eclipse’s Remote Application Platform (RAP) (http://eclipse.org/rap/). The main reason for that decision was the RAP’s small requirements on client machine capabilities and bandwidth of data connection. Using Eclipse RAP, any complex functionality is executed on the server.

### Repository

The repository component provides the functionalities needed to manage any persistent data in support of other system components, i.e. accessing, managing, versioning and (combined) querying for

- published annotations
- imported and annotated original records
- agent profiles and
- authentication and authorisation data.

AnnoSys uses the Resource Description Framework (RDF) (http://www.w3.org/RDF/) based W3C OA Data Model (http://www.openannotation.org/spec/core/2013 02 08/) for managing annotations in the AnnoSys Data Model (see below) and thus it needs a way to store RDF data. In principle, AnnoSys may connect to any RDF store compatible with the Apache Jena Framework (http://jena.apache.org/). After considering a series of evaluation reports and benchmarking studies (13) (http://wifo5-03.informatik.uni-mannheim.de/bizer/berlinsparqlbenchmark/results/V5/index.html) (http://wifo5-03.informatik.uni-mannheim.de/bizer/berlinsparqlbenchmark/results/V6/index.html) (http: //wifo5-03.informatik.uni-mannheim.de/bizer/berlinsparqlbenchmark/results/V7/index.html), AnnoSys uses Virtuoso Open-Source Edition (http://virtuoso.openlinksw.com/dataspace/dav/wiki/Main/) and Apache Jena TDB (http://jena.apache.org/documentation/tdb/) to store the published annotations and agent profiles, respectively. We use both Virtuoso and Jena TDB because we need Virtuoso to allow for SPARQL queries from the public and TDB to allow the storage of personal stores in specific user directories. This is needed to prevent unpublished annotations from being publicly searchable. In terms of AnnoSys, agents are either registered users or machine services with access rights. The imported and annotated original records are stored in the server’s file system using a directory/file based naming hierarchy according to persistent AnnoSys Identifiers (see below). Agent profiles consist of a TDB store for unpublished agent annotations and property files capturing agent preferences. Agents are also identified internally by AnnoSys Identifiers, so the files can be stored in the same file system as the original records. Finally, dedicated SQLite (http://www.sqlite.org/) databases are used on the server to either store security related information or agent subscriptions (see below).

### Security

The security component handles secure agent authentication, role based authorisation (14) and management of personal agent profiles.

Apart from AnnoSys’ general multitenancy design, all networked data transmissions are protected by the Hypertext Transfer Protocol Secure (15) to prevent identity theft or privacy invasion by wiretapping. The management of agent profiles combines the maintenance of the security database in general and of agent profile data including their repositories with respect to the requirements of the multitenancy design.

Authorisation in AnnoSys is built on permissions. These permissions may either be assigned to agents individually or grouped by role definitions (e.g. curator permissions for a given collection), which may then be assigned to agents. In particular, curator roles defined for institutions or collections have to be continuously updated with respect to the rights assigned to them: whenever new records or new record revisions have been created during repository import processes (e.g. new or updated record data submitted by a connected data portal), new (curator) permissions have to be created for the new record(s) and the respective role definitions must be updated. This is done automatically by the security component of the AnnoSys software.

All security functionality in AnnoSys, including access control, is based on the Apache Shiro framework (http://shiro.apache.org/), which is powerful, flexible, supports e.g. role based access control, and provides a programming interface that is easy-to-use (in comparison to, e.g. the Java Authentication and Authorization Services, JAAS) (http://docs.oracle.com/javase/7/docs/technotes/guides/security/ja as/JAASRefGuide.html). The security database, agent profiles including their repositories and all system relevant configuration files are held in a location on the server accessible only to authorised agents. For every registered user, Shiro creates an OA-agent in the RDF-store. Unless Shiro assigns the rights to publish annotations or to curate annotations in particular, there is no external write access to the annotation store.

### Services

To integrate AnnoSys in a data portal, the service component enables external services or web applications to integrate public data from the AnnoSys repository. There are no specific technical requirements to the portal. A detailed instruction on how to integrate AnnoSys with data portals is given in the AnnoSys technical documentation (http://wiki.bgbm.org/annosys/index.php/TechnicalDocumentation#Services_2). AnnoSys provides easy-to-understand RESTful web services (16, 17), Linked Data services (18) and SPARQL (http://www.w3.org/TR/sparql11-query/) endpoints.

The following request types are implemented by AnnoSys’ RESTful web services:

- list of all available annotations or records
- list of available annotations referring to a record with a given persistent AnnoSys Identifier (see below)
- list of available annotations referring to all records referring to a given UUID (see below)

In the response, all list entries are furnished with a persistent and resolvable URI identifier referring to the respective annotation or record. List entries are enriched by metadata information that may be used by data portals for display in their user interface.

The Linked Data service resolves the persistent URI Identifiers mentioned above and returns the referred annotation as an RDF (OA) document (e.g. https://annosys.bgbm.fu-berlin.de/AnnoSys/services/annotations/BGBM/An noSys/1400158295543.rdf) and the record data as an XML document (e.g. ABCD, https://annosys.bgbm.fu-berlin.de/AnnoSys/services/records/BGBM/AlgaTerra/3477/14 00158264774/abcd2.06b) on request, according to Linked Data principles.

The SPARQL endpoint allows external services to run self-defined SPARQL queries on the entire annotation repository.

The RESTful service returns information (i.e. annotation metadata like annotator’s name, annotation type, annotation date) in JSON (19) notation (e.g. https://annosys.bgbm.fu-berlin.de/AnnoSys/services/records/BGBM/AlgaT erra/3477/annotations, more details can be found in the technical documentation from http://wiki.bgbm.org/anno sys/index.php/TechnicalDocumentation#Request:_GET_.24 .7BServicesURL.7D.2Frecords.2F.3Clsid:authority.3E.2F. 3Clsid:namespace.3E.2F.3Clsid:objectId.3E.2F annotations). That way, data portals don’t need to know anything about OA or even RDF to integrate with the AnnoSys user interface and system. Because the quantity of metadata is not very high, no framing technique is provided.

RESTful and Linked Open Data services are implemented using the open Java API for RESTful Services (JAX-RS) (https://www.jcp.org/en/jsr/detail?id=339). The SPARQL Endpoint is realised using Virtuoso Open-Source Edition (http://virtuoso.openlinksw.com/dataspace/dav/wiki /Main/).

### Message system

The message system component aims at informing subscribing agents about changes in the annotation data. Messages are dispatched in AnnoSys via the user interface as well as by email to subscribers. The publication of annotations by annotators or curatorial annotations by curators via the AnnoSys user interface causes the message system to trigger notifications. Agents may subscribe to notifications according to the following topics:

- publication of (curatorial) annotations referring to records previously annotated by the given agent (automatically),
- publication of (curatorial) annotations referring to records within collections a curator has registered for (automatically) and
- publication of annotations matching a set of specific criteria (see Table 3) previously defined by a given subscriber.

In addition, if no curator is registered for a given record, the annotator can select contacts given in the record’s metadata and/or specify additional email addresses for notification.

The technical implementation of the message system builds upon the Java Message Service (JMS) (https://www.jcp.org/en/jsr/detail?id=914) and is implemented using Apache ActiveMQ (20) (http://activemq.apache.org/) as JMS Provider in tandem with Apache Camel (21) to realise internal and external message or email transports. A dedicated subscription database based on SQLite is used to store and manage agent subscriptions.

## The annotation context model and its application

### The generic annotation context model

Inspired by the SYNTHESYS annotation system (4), the first idea for the implementation of the annotation system was to use XML diff representations (22, 23) to capture the modifications conducted by an annotator in a second, annotated XML document. The visualisation of annotations could have been implemented based on XML diff results. Unfortunately, it turned out that XML diff result representations are highly complex with error-prone and low performing implementations. A second idea was the insertion of annotations as XML comments, linked to the annotated data elements within a given XML document. That approach was rejected due to severe conceptual difficulties like addressing XML attributes and sophisticated search operations in XML documents. Ultimately, the idea of realising some kind of ‘recorder’ functionality capturing the modifications applied to data elements when edited by annotators lead to the development of a generic annotation context model.

As a result, AnnoSys’ annotation context model pursues a completely generic approach. If an application depicting a data record is able to provide a schema-based on-line representation of the record, it can be connected to a system using the AnnoSys model. Using natural history collection data and the data standards used in that community is but one specific application of the generic model. The AnnoSys annotation context model builds upon the following cornerstones:

- Universally Unique Identifiers (UUIDs) (24) identifying the object
- a structured set of metadata describing individual objects referenced by UUIDs (annotated record)
- element selectors unambiguously designating the data element within a structured metadata representation that is subject to an annotation (annotated element)

Principally, this approach is agnostic with respect to data formats and standards as it only relies on structured metadata that somehow accommodate element selectors or filtering. However, schema based relational databases or data formats like XML or RDF are preferred in order to enable the implementation of selection mechanisms based on standard query or filter languages like SQL (25), XPath (http://www.w3.org/TR/xpath20/) or SPARQL (http://www.w3.org/TR/sparql11-query/).

**Figure 5.**
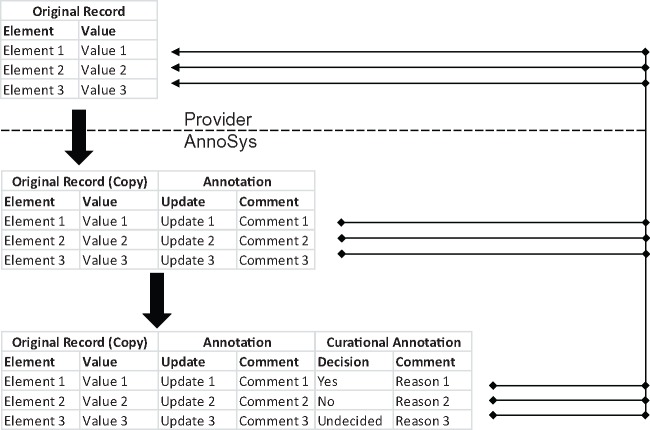
Data enrichment within the AnnoSys workflow.

Using this generic approach, an annotation context can be defined as a set of annotated elements referring to a given set of metadata and being descriptive of a distinct physical or virtual object belonging to a given data collection or database. In cases where annotations represent a corrected or new value for the element, they may be used to create new (quality enhanced) records for the object. Depending on the application use case, the annotated elements may be further enriched by information potentially triggering or supporting data maintenance tasks on the part of collection holders (see ‘Messaging System’ above). This information may include, e.g. value correction proposals, natural language comments or expectations (26) expressed towards the curator of the collection. Stated expectations may be to update, add, or remove the selected record data element of the annotated record. A curator can then decide to follow or reject the recommendation (Figure 5).

The model also allows for the annotation of multiple elements within a single record. We identified several use cases in the biodiversity domain for this. For example, the annotation of geographic information such as longitude and latitude in itself already refers to two data elements and potentially impacts on other location-based information like country or region names.

### UUIDs—a requirement not easily met

As the current AnnoSys implementation requires structured collection metadata to be accessible as XML documents, AnnoSys implements XPath expressions as element selectors.

In the context of the organisation for Biodiversity Information Standards (TDWG) (http://www.tdwg.org/), the biodiversity research community has developed metadata standards for physical specimens in collections, particularly the aforementioned Darwin Core (27) and ABCD (http://www.bgbm.org/TDWG/CODATA/Schema/) schemas. The reference to the physical object is made by the so-called ‘triple identifier’, which is supposed to uniquely identify a specimen—i.e. to function as a globally unique identifier (GUID (http://www.tdwg.org/activities/guid/) (http://www.tdwg.org/standards/150/)). The AnnoSys ‘tripleId’ consists of three data elements, designating (i) an individual specimen object (unit) belonging to (ii) a determined collection which is physically located at a (iii) given institution (e.g. a herbarium). Several issues have been recognised for some time with this approach, and accordingly, stakeholders such as GBIF recommend the use of OccurrenceId to stabilise the reference. However, this term in itself is ambiguous, because in the case of specimens this may refer to a specific collection event [a ‘field unit’(28)] and not a specific specimen. The introduction of unique resolvable identifiers by some of the major international collection institutions may eventually lead to resolving this problem for specimens (29).

The principal problem with this approach is that the tripleId is essentially designating the collection object, not the metadata describing the object. Although the object itself is (usually) quite stable, the metadata may change (e.g. by the incorporation of corrections to the metadata resulting from an annotation process). The tripleId and the respective XML documents do not provide a clearly defined mechanism to identify such versions of the metadata records. Moreover, one and the same physical object may be described in different metadata records (e.g. one coming from the collection itself, and one coming from literature citing the object). To overcome this problem, AnnoSys decided not only to store the respective current version of the metadata record with every annotation, but also to extend the tripleId by developing the AnnoSys Identifiers.

### Persistent AnnoSys identifiers (AnnoSysIds)

As described earlier, when a user starts an annotation, an XML record document is retrieved by AnnoSys. Before the record gets annotated, this document will be analysed and compared for similarity to former revisions of XML documents already archived within the repository for the same tripleId and document format. Two documents are considered to be ‘similar’ if they contain the same elements and attributes (including values) regardless of order. If no similar record exists within the repository, the retrieved record document is added to the repository, becomes the most recent revision for the given tripleId, and will be related to the annotation actually being created. If a ‘similar’ archived XML document revision exists, then this revision will be related to the annotation. If a dissimilar archived document with the same tripleId exists, this becomes a ‘historic’ revision, which cannot be annotated anymore. This workflow has been implemented because any changes at the source of the XML record identified by a given tripleId may potentially invalidate previous annotations. Although the version history of annotations referring to a historic metadata record revision will also be shown in the user interface, only the most recent one can be annotated.

By extending the notion of tripleIds with version information AnnoSys always relates annotations to a specific revision of an XML record document.

Although record data are uniquely identified by a tripleId, they may be provided in different data formats (e.g. ABCD or Darwin Core). Therefore, within the annotation model each annotation is related to the specific schema. Otherwise, annotations could not be correctly reproduced due to different XPath selectors. To support that, AnnoSys further extends the tripleId notion by adding a defined namespace prefix for each supported XML data standard (see Table 4).

**Table 4.** Components of tripleId, LSID and AnnoSys Identifier.

Name	Content	TripleId	LSID	AnnoSysId
Institution identifier	Institution code	yes	yes	yes
Collection identifier	Collection code	yes	yes	yes
Unit identifier	Catalogue number	yes	yes	yes
Version	Revision	no	Yes	yes
Format	Namespace prefix	no	no	yes

Although *BGBM:Herbarium+Berolinense:B+18+0014862:1379406965371:abcd2.06b* is an example for a record with the tripleId *BGBM:Herbarium+Berolinense:B+18+0014862*, imported at 17 September 2013, 08:36:05 (version is stored as time in millisecond since 1 January 1970, 00:00:00) in ABCD data format version 2.06b (namespace prefix is abcd2.06b), *BGBM:AnnoSys:1379006965559* is an example for annotation created by AnnoSys. Annotations do not require version and format information, because they are only created once and are always stored in compliance to the OA Model.

### The annotation data model of the current AnnoSys implementation

**Figure 6.**
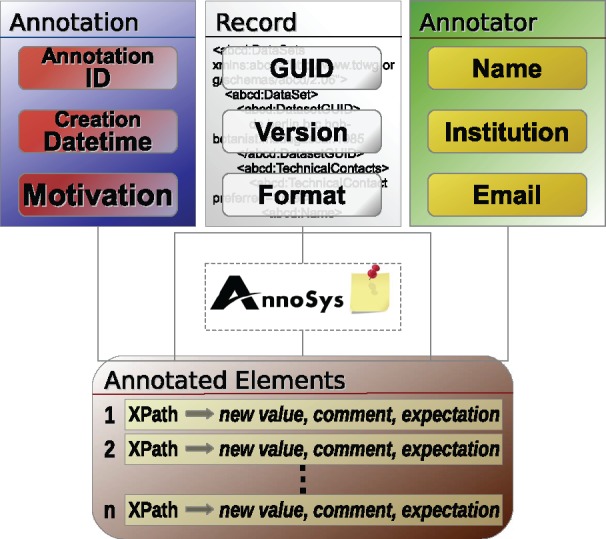
Annotation data model of the current AnnoSys application.

The current AnnoSys data model is an implementation of the Generic Annotation Context Model, based on annotation metadata, metadata about the annotated records, metadata about the annotator and a set of annotated elements (Figure 6).

The main metadata element of the annotation is the AnnoSysId. For each annotation created, an AnnoSysId is generated and assigned by the system. In analogy to the tripleId of the specimen, the AnnoSysId includes a designation of the organisation, the system and the individual record. This makes it possible to distinguish annotations created or imported from different annotation systems or AnnoSys instances (e.g. BGBM:AnnoSys:123456). Furthermore, annotation metadata include a motivation (annotation type, see Tables 3 and 6) and the annotation’s creation datetime.

The metadata on the annotated record consists of the record’s AnnoSysId, which allows a client to seamlessly access the corresponding record metadata from a data provider or via the repository component.

The annotator’s metadata include the name, institution and email address of the annotating agent. Although the email address is not public, the publication of name and institution follows the scientific practice in biodiversity collections.

Finally, annotated elements represent a list of record data elements selected by an annotator that relate element selectors with additional information about the addressed element within the annotated record. As the current AnnoSys implementation works with XML data, element selectors are expressed as XPath expressions. Element selectors addressing a document’s root element (‘/’) are used to express general comments about the entire annotated record. Beside the element selector, an annotated element consists of an expectation expressing the expected corrective to be taken by the curator with regard to the annotated data record in the collection database (see Table 5). Optionally, the additional information includes an annotated value and/or a comment. Therein, the annotator may propose a new value for the selected data element and/or make natural language comments for the selected element.

**Table 5.** Supported expectations.

Expectation	Description
Add	Add new element according to the element selector to annotated data record
Remove	Remove element from annotated data record
Update	Update element in annotated data record

Besides regular annotations created by annotators, AnnoSys’ annotation data model must also cover the concept of curatorial annotations (see Figure 6). In contrast to regular annotations, curatorial annotations annotate published annotations, thus they are annotations of annotations. Their motivation (30) (annotation type) is fixed to the value ‘Curatorial’ (see Table 6). Furthermore, the term ‘curated element’ is introduced in place of ‘annotated element’ and a list of curated elements replaces the list of annotated elements known from regular annotations. The additional information included in curated elements consists of the curatorial decisions taken by a curator regarding the expectation and optional values provided by a given annotated element (see Table 7). Optionally, a natural language comment may be submitted to provide a more detailed, verbatim justification of the curator’s decision.

**Table 6.** Meta annotation types.

Annotation type	Content	Elements involved
Curatorial	Meta annotation referring annotations commented on by a curator. Each annotated value can be accepted, rejected and discussed	All elements of the curated annotation
Batch	Meta annotation linking identical annotations referring to different annotated records	All elements of the linked annotation(s)
CuratorialBatch	Meta annotation referring to all curated annotations of a Batch and its annotation type. Each annotated value can be accepted, rejected or commented by the curator for all annotations linked by the referred Batch	All elements of the curated BatchAnnotations

**Table 7.** Supported decisions in curatorial annotations.

Expectation	Description
Accepted	Element accepted by curator and updated in collection database
Rejected	Element rejected by curator and not updated in collection database
Undecided	Further processing of element in collection database not yet decided
Update	Element already updated for another reason

**Figure 7.**
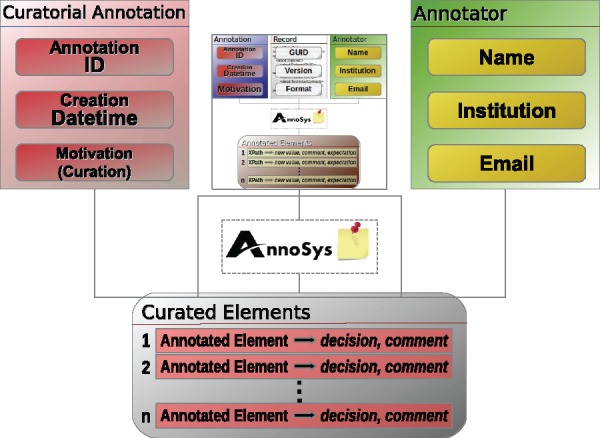
Curatorial annotation data model.

**Figure 8.**
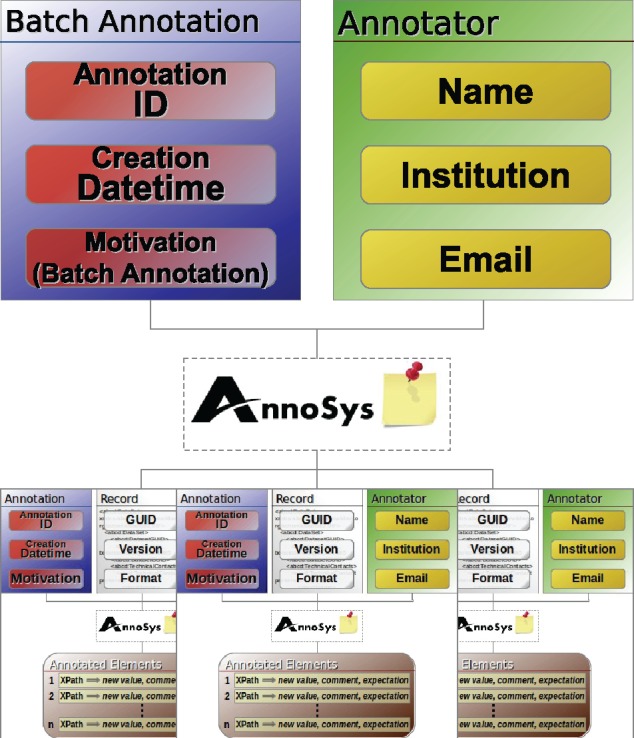
Batch annotation data model.

Finally, the AnnoSys annotation data model has to provide means to support batch annotations. Each individual annotation of a batch annotation will be stored in the repository like any other regular annotation. In order to link these annotations together, a further annotation with an annotation type of either ‘Batch’ or ‘Curatorial Batch’ will automatically be added (see Table 6). Annotated or, respectively, curated elements (Figures 6 and 7) become obsolete, instead, the metadata on the annotated record are replaced by a list of references to annotations affected by the given batch annotation (Figure 8).

## OA-based implementation of the AnnoSys data model

The activities of the W3C OA Community Group (https://www.w3.org/community/openannotation/) significantly alleviated the decision process towards a suitable storage and exchange format for annotations. Another reason for choosing OA was that the system requirements for AnnoSys include maximal genericity and flexibility for future changes and extensions, e.g. transposition to a completely RDF-driven data scheme to allow the integration with other RDF-based knowledge bases or to extend semantic queries to annotated record data. RDF features, such as facilitating data merging even if the underlying schemas differ, or to support the evolution of schemas over time, are needed to meet these requirements. Furthermore, providing public read access to the entire annotation knowledge base (repository) via a SPARQL endpoint enables external services to define and run more sophisticated and perhaps experimental queries than those offered by the AnnoSys web services.

The W3C OA Community Group pools the earlier initiatives Annotation Ontology (30) and OA Model (http://www.openannotation.org/spec/beta/). The consolidation of both initiatives under the umbrella of W3C increases the prospects for long-term sustenance of the upcoming standard. W3C OA ‘specifies an interoperable framework for creating associations between related resources, annotations, using a methodology that conforms to the Architecture of the World Wide Web’ (http://www.openannotation.org/spec/core/index.html). It provides a non-normative OWL ontology (https://www.w3.org/ns/oa#), so that any kind of URI resolvable web resource may be used as a target for an annotation.

Although the first published specifications W3C OA Core Data Model (Community Draft, 9 May 2012) (http://www.openannotation.org/spec/core/20120509/) and W3C OA Extension Specification (Community Draft, 9 May 2012) (http://www.openannotation.org/spec/extension/2 0 1 20509.html) provided powerful means to express AnnoSys’ basic requirements, AnnoSys’ first prototype implementation revealed some weak points and limitations preventing a semantically correct and specification compliant transposition of the AnnoSys data model. Subsequently, the presentation of the AnnoSys use case and following discussions within the community group yielded similar problems in other projects (https://lists.w3.org/Archives/Public/public-openannotation/2012Aug/003 7.html) (https://lists.w3.org/Archives/Public/public-ope n a nnotation/2012Oct/0004.html) (https://lists.w3.org/Archi v es /Public/public-openannotation/2013Jun/0004.html). This led to an upgraded specification W3C OA Data Model (Community Draft, 8 February 2013) (http://www.openannotation.org/spec/core/20130208/index.html) in response to the demands defined by AnnoSys and other projects. AnnoSys’ repository implementation is based on that revised specification.

Meanwhile, OA stepped up and served as a home base for the W3C Web Annotation Working Group (http://www.w3.org/annotation/), which currently elaborates W3C Candidate Recommendations (version 06 September 2016) for a Web Annotation Data Model (http://www.w3.org/TR/annotation-model/), Web Annotation Vocabulary (https://www.w3.org/TR/annotation-vocab/) and Web Ann otation Protocol (https://www.w3.org/TR/annotation-protocol/). The working draft enhances the former OA Data Model used by the AnnoSys implementation by introducing some new modeling elements while clarifying and redefining more details of the former data model. Beyond that, the Web Annotation Protocol provides standardised methods to exchange and synchronise annotations between different annotation repository providers. Within the following sections, the technical transposition of AnnoSys’ data model into the W3C OA Data Model will be described.

### Basic annotation

Principally, there are two alternatives for implementing complex data models based on the W3C OA Data Model. First: use basic OA features only to identify a target resource (versioned record data) and to formalise a domain-specific ontology for describing annotations within the body. Second: make use of all OA features in order to completely express annotations by means of OA only. Within AnnoSys, we decided to use the second alternative in order to provide a solution as close as possible to the OA standard, and thus become applicable to non-biodiversity domains as well.

**Figure 9.**
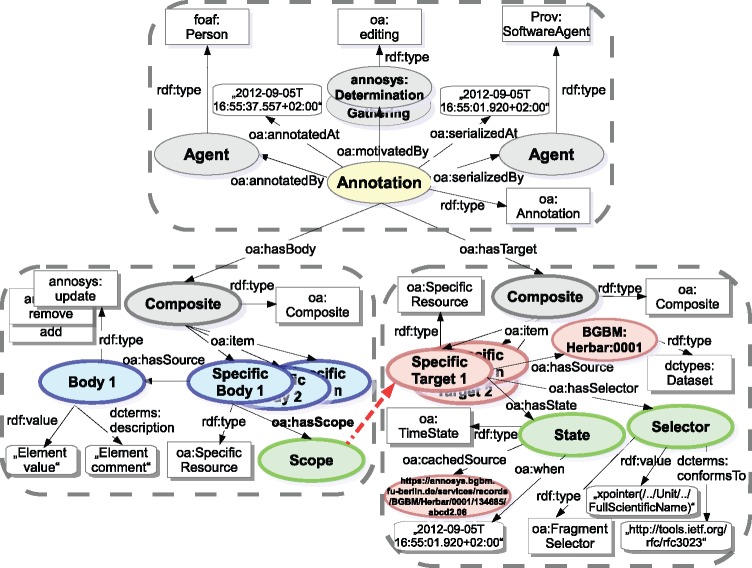
Open Annotation implementation of the AnnoSys Annotation Data Model.

Basically, an annotation created with AnnoSys according to the W3C OA Data Model (Community Draft, 8 February 2013) includes the following main parts (see Figure 9):

- provenance (required, upper frame)
- target (required, frame connected via oa:hasTarget relation) and
- body (optional, frame connected via oa:hasBody relation)

The provenance part holds basic annotation metadata like annotator information, annotation creation datetime, annotation type (oa:motivatedBy) and information about the generating software instance, generation datetime and generated model version.

The target part contains a description of the annotated target resource. Apart from a URI identifier of the targeted resource, it cites state information (e.g. resource retrieval datetime, location of cached resource copies) about the referenced target resource and/or a data element selector into it.

The body part includes a comment or another resource stating something about the target.

Annotated elements are realised by a pair of specific body and target resources directly related to the corresponding annotation resource. Thereby, the scope relationship (oa:hasScope) permits to denote a soft linking between a body (holding annotated value and comment) and a target (specifying element selector and record metadata). Semantically, OA is liable to the open world assumption, so its semantic interpretation with regard to multiple bodies and targets may be expressed as ‘all bodies of an annotation apply to all targets of an annotation’. That is, any combination of annotated value/comment pair specified within a body relates to any pair of element selector/record metadata. As this is not the intended semantic meaning of annotated elements described in AnnoSys’ data model, the scope relationship is used to establish a context between interconnected (specific) body and target resources in order to describe the common bond (or reference) between annotated data and the related annotated element within a record. It could be translated into natural language like ‘the scope denotes the selected data element(s) within a source document the annotator examined while generating the given pair of annotated value and/or comment’ (https://lists.w3.org/Archives/Public/public-openannotation/Mar/0030.html, https://lists.w3.org/Archives/Public/public-openannotation/2013Mar/0031.html, https://lists.w3.org/Archives/Public/public-openannotation/2013Mar/0038.html, https://lists.w3.org/Archives/Public/public-openannotation/2013Mar/0040.html). That way, the open world assumption contradicting hard links between specific body and target resources could be resolved by soft link relations.

The OA’s Fragment Selector class allows for the definition of a standard the selector expression conforms to. With regard to the implementation of AnnoSys’ element selectors XPointer (http://www.w3.org/TR/xptr-framework/) perfectly match these requirements by enabling the expression of XPath compatible selectors within XML documents and thus allowing the selection of parts of the record. Within the user interface, the fragment selector is reflected in the specific entry fields. Each field represents one element of the ABCD/DwC-schema, i.e. specifies a part of a record. The corresponding XPath to each element or entry field, respectively, is shown in the tool-tip of each field.

Annotation types (determination, gathering etc.) can be perfectly expressed by the oa:motivatedBy properties in an OA annotation's provenance part. Although the oa:motivatedBy property expects instances of the class *oa:Motivation*, OA provides a set of predefined subclasses to be extended by applications. As the basic expectation of annotators towards a collection curator is to edit or update the collection database based on the published annotation, subclasses reflecting AnnoSys annotation types (e.g. annosys:Determination, annosys:Gathering, see Table 3) have been derived from subclass *oa:editing*.

Tables 8 and 9 show the direct mapping of AnnoSys’ annotation metadata and annotated element specifications into the OA Data Model.

**Table 8.** Direct mappings from AnnoSys' annotation metadata into the updated OA Data Model.

AnnoSys data model	OA part	OA property
Motivation	Provenance	oa:motivatedBy
Datetime (of the creation)	Provenance	oa:annotatedAt
Annotation GUID	Provenance	annotation resource uri
Annotator	Provenance	oa:annotatedBy
Record GUID	Target	oa:hasSource
Record document format	Target	oa:cachedSource (from oa:hasState)
Record document version	Target	oa:when (from oa:hasState)

**Table 9.** Direct mappings from AnnoSys annotated elements to the updated OA Data Model.

AnnoSys data model	OA part	OA property
Element selector	Target	oa:hasSelector
Expectation	Body	rdf:type
Annotated value	Body	rdf:value
Comment	Body	dcterms:description

### Curatorial annotations

**Figure 10.**
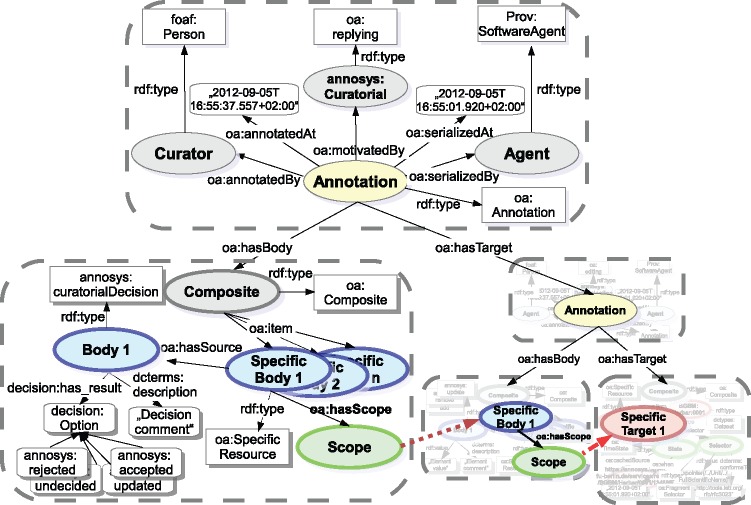
W3C Open Annotation implementation of AnnoSys Curatorial Annotations.

A new motivation class called *annosys:Curatorial* has been introduced, with subclass *oa:replying* reflecting the intended character of curatorial annotations. The provenance part remains unchanged compared with the modeling of regular annotations. As OA defines that annotations must include at least one target, the target of a curatorial annotation now references the annotation it comments upon. The relationship between any curated element of a curatorial annotation and the corresponding annotated element of the annotation provides an additional link. The relationship is established via the specific body resource of the element of the curatorial annotation which references the corresponding body resource of the annotated element via the oa:hasScope relationship. In combination with the decision modeled within the body resource based on the *Decision* class taken from the decision-ontology (https://code.google.com/p/decision-ontology/) and a decision comment attached via the specific body's *oa:hasSource* relation, a curated element instance is depicted in Figure 10.

Table 10 depicts the mappings from AnnoSys’ curated elements into OA’s Data Model. Curatorial processing types have been subclassed from the decision-ontology’s class *decision:Option* in order to reflect the curator decision types defined by AnnoSys Data Model (e.g. annosys:accepted, annosys:rejected, see Table 7).

**Table 10.** Direct mappings from AnnoSys' curated elements into OA's Data Model.

AnnoSys data model	W3C OA part	W3C OA property
Annotated element	Specific body	oa:hasScope
Curational processing	Body	decision:hasResult
Comment	Body	dcterms:description

### Batch annotations and batch curatorial annotations

**Figure 11.**
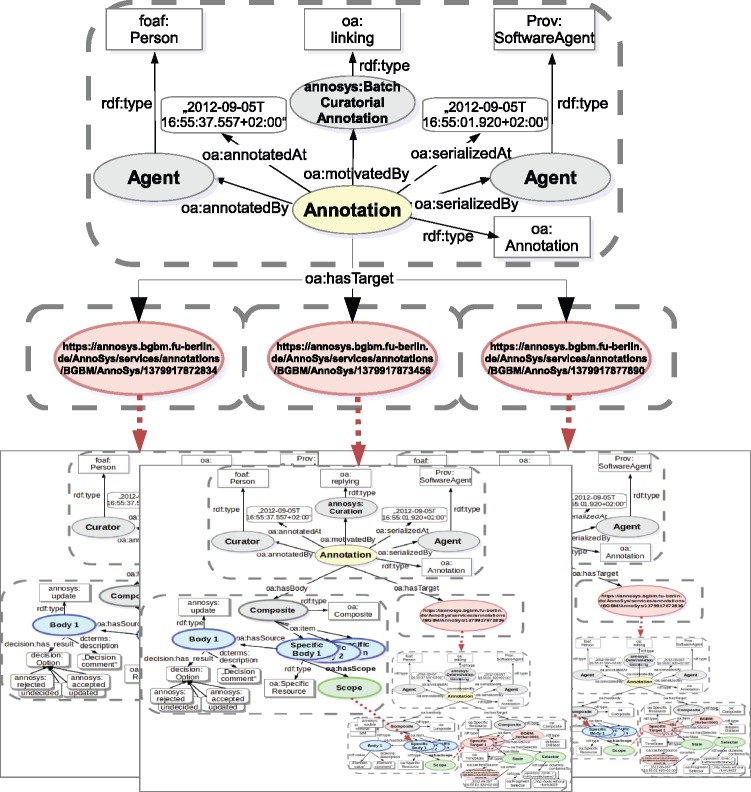
W3C Open Annotation implementation of AnnoSys Batch Annotations.

Batch annotations are designed to link together a set of identical annotations or curatorial annotations referring to distinct data records (annotations) identified by their resource URI’s (Figure 11).

The batch annotation’s metadata part remains mostly unchanged as compared with a simple annotation. The motivation classes annosys:BatchAnnotation and annosys:BatchCuratorialAnnotation have been introduced and derived from oa:linking to reflect the nature of batch annotations. In comparison to the more general concept of Annotation Sets or Collections, grouping any kind of annotations as specified in the Annotation Ontology (https://code.google.com/archive/p/annotation-ontology/) or OA in EPUB (http://www.idpf.org/epub/oa/) drafts, the content of any AnnoSys annotation contained in a batch is identical, but each of them references a different target (record for batch annotations, annotation for batch curatorial annotations).

Usually, Batch Annotations have no body but refer to the enveloped annotations by including multiple targets referring to the enveloped annotations’ resource URIs.

## Discussion

A generic shared annotation system across multiple data sources and portals offers the opportunity for the entire community to pool expertise and benefit from the contributions of all parties. AnnoSys provides a solution by offering a user interface that automatically generates annotations in a standardised format, providing generalised annotation body structures and generalised robust fragment selection mechanisms and identity management by role based authorization. Portals that can provide structured data records need no further testing and validation of application level interfaces to integrate AnnoSys, except adding an ‘Add annotation’ button and a list of already existing annotations. Future releases of AnnoSys will provide legacy versions of web service API functions in order to guarantee stability across software versions and avoid untying data portals or services currently connected to the system. Also, AnnoSys is resilient to unrelated changes to data because it ensures that always the latest version of a record as retrieved from the data provider is annotated. However, although these requirements are met, encouraging the readiness of users to register and contribute annotations is a long lasting process. Prerequisites are a high profile of AnnoSys within the community and the motivation of users to become part of a networked community by contributing to data quality and enrichment. Therefore, scientists working with specimens need to be more directly addressed, as well as individual institutions holding specimen data. Critical stakeholders that help to increase the number of contributions are therefore individual institutions that integrate AnnoSys into their collection web pages and their workflows, as well as data aggregators like GBIF, JSTOR or Europeana. The cooperation with citizen science projects may also be a further step to motivate users to share their expertise and contribute annotations.

### A generic system

AnnoSys was originally tailored for annotation workflows in virtual herbaria (3), but the present AnnoSys implementation is generic for all kinds of biological collection data (e.g. zoological natural history collections, *exsitu* collections in botanical and zoological gardens), as well as for species occurrence observation data on all kinds of organisms. Potentially, the complete content of the rapidly growing BioCASe and GBIF networks (649 Mio records as of 31 May 2016) (http://www.gbif.org/) can be annotated via AnnoSys. This domain is a particularly challenging one for the development of an annotation system, because the actual data of the objects potentially change over time at the source [in contrast to, say, archival documents or published literature, as e.g. dealt with in SharedCanvas (11)].

The generic approach makes AnnoSys adaptable to purposes beyond biological collections. Logical extensions within the biodiversity domain are for example annotations of specimen images, or annotations of taxonomic database records, such as those in checklists (e.g. Euro + Med PlantBase [http://ww2.bgbm.org/EuroPlusMed/) and Fauna Europaea (31)]. Generally, AnnoSys is suitable for schema-based data about real world objects having digital representations (32), which can be enriched with corrections, additions published after databasing, or new scientific content. These objects can be physical materials from archives or museums as well as printed material and artwork in a broad sense. To widen the scope, AnnoSys must be adapted to support the respective data schema and a domain-specific interface must be implemented.

### Strategies for enhancing data quality

AnnoSys is all about enhancing data quality. This is a cross-sectional topic in data networks in science, affecting generation, publication, distribution, and usage of data (32). In data management and publication, we see two main strategies for handling data quality.

The first strategy for the handling of data quality issues is for the aggregator to filter for erroneous data and (where possible) correct them before publication. This strategy is used inter alia by GBIF. GBIF do not own the data provided by different data publishers and institutions, i.e. they have no access to original data bases and are presently not able to directly inform data providers about each data quality issue (although issues detected are communicated on the GBIF.org site). On the one hand, this is minimizing the publication of erroneous data (misspelled scientific names, misplaced geo references etc.) and it raises the quality level of standardised data. On the other hand, errors in the original data sources are not corrected and data which are not compliant to the authority files used might be difficult to find or be omitted by the system (e.g. scientific names that are newer than those used in the names backbone). In the GBIF portal, these corrections are handled independently from a rudimentary annotation system (‘feedback’), which allows to provide a simple comment on the record displayed.

The second strategy aims at correcting the original data. All standards (http://www.tdwg.org/) and manuals for handling data quality (33) are helpful for data preparation and data correction at the source. For existing data, services may help to find errors in individual databases (34). Annotations are the way to improve data already published.

An approach taken by JSTOR Global Plants (http://plants.jstor.org/) is the inclusion of a forum for feedback on specimen records, with the aim of encouraging curators to update their data. The advantage is the low barrier for scientists to contribute, the disadvantage of this system is that the information is unstructured and lacks differentiation between data corrections, reasons for corrections and contributions to evaluation or interpretation. Moreover, the annotations are only visible in the JSTOR data portal, and annotations made in other portals, many of which share the specimen data displayed in JSTOR, are not accessible. To overcome these shortcomings, the forum is planned to be complemented by AnnoSys.

The Filtered Push (26, 35) approach is to ‘push’ annotations created at connected platforms or data portals into a network of consuming nodes. Further annotation processing is up to the consuming node, but actually the common use case is supporting data curators by suggesting corrections of data elements in their individual collection databases. Data are structured and the purpose of annotations is made explicit. In this approach, annotations are not accessible in a central repository. This has several disadvantages: (i) annotations are not accessible to interested users outside the network of collections; (ii) connected portals cannot retrieve annotations, which may lead to the creation of redundant annotations pushed into the network (additional workload for curators); and (iii) annotations of fixed source data (e.g. compilation of specimens from literature sources) are futile because they will not have any effect.

The AnnoSys approach also aims at correction of original data, but (i) it makes published annotations openly accessible and users may subscribe to the specific topics they are interested in, (ii) potentially feeds back existing annotations to any portal that is able to resolve the respective object identifier (see Table 1 for current implementations) and (iii) stores all annotations (i.e. also those referring to fixed sources) for subsequent use. In addition, AnnoSys is able to handle complex schemata and thus supports domain-specific structures, such as multiple taxonomic identifications or the assignation of modern geo reference vs. historical place names. AnnoSys also allows registered curators to evaluate annotations of specimens from their collections, and to publish their response to annotations, making the arguments visible and transparent for the public. The results do not necessarily end up in an update of their collection database, but nevertheless may be an important contribution to data quality. These advantages do come at the price of additional complexity, but offer opportunities for a much wider application of the system.

Notifying data providers is an important element when aiming at data quality enhancements at the source. Earlier evaluations (7) have shown that notifying data publishers may result in the correction of original data in databases within hours, but may also have no effect at all. The latter may be the case when datasets are orphaned, not curated, not updated because of technical reasons, or if updating is undesirable because they are stored as stable research results (36). For data publishers interested in updating, AnnoSys refined the messaging system used by SYNTHESYS (4) by including subscriptions and textual data display.

AnnoSys is as far as we know the only system aiming at correcting original data by notifying data providers as well as providing the annotations to portal users, alerting them that the displayed data may include data quality issues. On top of that, potential data quality issues are spread to all people interested in a certain topic by means of subscriptions.

## Outlook

Even though the presented AnnoSys data model is generic with respect to any kind of XML Schema-based standard format, the currently available release version only supports ABCD and DarwinCore based record documents. The extension of AnnoSys to support a wide range of existing and planned online data systems is thus a top priority. In particular, supporting the various record formats (RDF, text, XML) of the widely distributed DarwinCore standard is needed to completely cover the biodiversity domain [including, e.g. the Global Genome Biodiversity Network (GGBN) (37)]. For this, the AnnoSys data model will be reviewed with regard to the element selector specification, which is heavily used in predefined annotation type templates. This needs to be adopted to simultaneously support semantic concepts as well as static XML schemas. This is also a pre-requisite for enabling interoperability between AnnoSys and other annotation systems [e.g. FilteredPush (26) (http://wiki.filteredpush.org)]. Another area for future development is supporting the annotation of (specimen) images. OA already supports this by means of area selectors. However, to follow the AnnoSys model we need to store a representation of the original image. To be able to process the high-resolution image formats typically used for specimens, smart solutions with regard to version archiving and transmission performance will have to be researched in order to include image annotation in AnnoSys. Last not least, the messaging system should be improved to support protocols like RSS feeds or Twitter.
