# AnnoSys—implementation of a generic annotation system for schema-based data using the example of biodiversity collection data

**DOI:** 10.1093/database/bax036

**Published:** 2017-05-06

**Authors:** L. Suhrbier, W.-H. Kusber, O. Tschöpe, A. Güntsch, W. G. Berendsohn

**Affiliations:** Botanic Garden and Botanical Museum, Freie Universität Berlin, Königin-Luise-Str. 6-8, 14195 Berlin, Germany

Several errors were noted in the above paper after publication and have now been corrected.

In section ‘Message System’, the link to Table 3 has been replaced by a link to Table 2.

In section ‘OA-based implementation of the AnnoSys data model’:

* The link to Figure 6 in ‘the concept of curatorial annotations (see Figure 6)’ has been replaced by a link to Figure 7.

* The link in ‘methodology that conforms to the Architecture of the World Wide Web (http://www.openannotation.org/spec/core/index.html)’ has been corrected

In the Acknowledgements section, the hyperlink to http://api.gbif.org/v1/ has been replaced by "http://www.gbif.org/developer"

Several other minor corrections have also been made that do not affect the academic content of the work, including several malformatted URLs that have all now been presented correctly.

The original article has now been updated to reflect the changes. The authors apologise for these errors.

